# Diagnostic Efficacy of Plasma-Based Real-Time PCR for Schistosomiasis Japonica in Mice before and after Treatment with Praziquantel

**DOI:** 10.3390/ani13193068

**Published:** 2023-09-29

**Authors:** Cheng Chen, Xue Zhou, Qinghong Guo, Chao Lv, Yalan Tang, Qingqing Guo, Yang Chen, Kerou Zhou, Zhiqiang Fu, Jinming Liu, Jiaojiao Lin, Yang Hong, Jun-Hu Chen

**Affiliations:** 1 National Reference Laboratory for Animal Schistosomiasis, Key Laboratory of Animal Parasitology of Ministry of Agriculture and Rural Affairs, Shanghai Veterinary Research Institute, Chinese Academy of Agricultural Sciences, Shanghai 200241, China; 2 National Health Commission of the People’s Republic of China (NHC) Key Laboratory of Parasite and Vector Biology, National Institute of Parasitic Diseases, Chinese Center for Diseases Control and Prevention (Chinese Center for Tropical Diseases Research), World Health Organization (WHO) Collaborating Center for Tropical Diseases, National Center for International Research on Tropical Diseases, Shanghai 200025, China; 3 Hainan Tropical Diseases Research Center (Hainan Sub-Center, Chinese Center for Tropical Diseases Research), Haikou 571199, China

**Keywords:** *Schistosoma japonicum*, real-time fluorescent quantitative PCR, praziquantel, efficacy evaluation

## Abstract

**Simple Summary:**

Molecular diagnostic methods based on nucleic acid detection possess more advantages in high sensitivity and specificity, and low cross-reactivity with other pathogens. We assessed the detection efficacy of a real-time fluorescent quantitative PCR assay for schistosomiasis japonica in mice, before and after treatment with praziquantel. The sensitivity of the method was 99.3% (152/153, 95% CI: 96.41–99.98%) and its specificity was 100% (77/77, 95% CI: 95.32–100%). The results showed that the method exhibited good sensitivity and specificity, and its sensitivity correlated with the infection intensity in mice. After the oral administration of praziquantel, mice infected with 10 cercariae or 40 cercariae were all *Schistosoma japonicum*-negative via this method 6 weeks after treatment. This method had advantages over a soluble-egg-antigen-based enzyme-linked immunosorbent assay, and possessed better potential utility for evaluating the treatment efficacy of praziquantel in schistosome-infected mice.

**Abstract:**

The prevalence of schistosomiasis japonica in China is now characterized by a low epidemic rate and low-intensity infections. Some diagnostic methods with high sensitivity and specificity are urgently needed to better monitor this disease in the current situation. In this study, the detection efficacy of a real-time fluorescent quantitative PCR (qPCR) assay was assessed for schistosomiasis japonica in mice, and before and after treatment with praziquantel (PZQ). Our results showed that the sensitivity of the qPCR was 99.3% (152/153, 95% CI: 96.41–99.98%) and its specificity was 100% (77/77, 95% CI: 95.32–100%) in mice infected with different numbers of *Schistosoma japonicum.* After the oral administration of PZQ, mice infected with 10 cercariae or 40 cercariae were all *Schistosoma japonicum*-negative 6 weeks after treatment. However, the negativity rates on a soluble egg antigen (SEA)-based enzyme-linked immunosorbent assay (ELISA) were only 34.8% (8/23, 10 cercariae group) and 6.7% (1/15, 40 cercariae group) at the sixth week after PZQ treatment. These results demonstrated that the qPCR method had good sensitivity and specificity, and suggested that its sensitivity correlated with the infection intensity in mice. Moreover, this method had better potential utility for evaluating the treatment efficacy of PZQ in schistosome-infected mice than SEA-based ELISA.

## 1. Introduction

Schistosomiasis is a serious zoonotic parasitic disease in humans, caused by three species of the blood fluke *Schistosoma*. It is distributed in 78 tropical and subtropical countries and areas, and affects more than 230 million people worldwide [1]. In China, schistosomiasis japonica, caused by *S. japonicum*, is a major public health problem and was once epidemic along the Yangtze River, infecting around 12 million people. With 70 years of continuous national control programs, the prevention and control of schistosomiasis in China have made remarkable advances. Schistosomiasis japonica has been eradicated in many previously disease-endemic regions, and the prevalence of the disease is now characterized by a low epidemic rate and low-intensity infections [2].

Traditional parasitological diagnostic techniques, which rely on the detection of schistosome eggs, miracidia, or worms are still the “gold standard” methods. Immunodiagnostic techniques are also widely used in endemic areas to detect schistosomal antibodies in their definitive hosts. These techniques, which are more sensitive and easier to perform than traditional methods, have become commonly used monitoring tools in many schistosome-endemic areas. In China, both parasitological and immunological methods are included in the national schistosomiasis control program. However, some shortcomings and challenges of these methods began to appear in the current epidemic, in which the infection rates and infection intensities in endemic areas were lower than in previous epidemics [2,3]. For example, parasitological diagnosis showed poor sensitivity for early or low-intensity infections, although it was very suitable for field testing because it was inexpensive and simple to operate. The main limitations of immunodiagnostic techniques are the false-positive results when the antibodies used crossreact with other parasitic diseases and keep a long time in evaluation of the efficacy of chemotherapy. Furthermore, other animals, including domestic animals, must also be monitored for infection and the lack of appropriate commercial secondary antibodies limits the use of immunodiagnostic techniques [4,5,6,7,8].

Molecular diagnostic methods based on nucleic acid detection have become more frequently used in the diagnosis of parasite diseases in recent years. With the development of genomics and the publications of genome data for *Schistosoma*, several molecular targets have been identified and several molecular assays can detect schistosome DNA in a variety of samples. These detection methods exhibit higher specificity and sensitivity and less crossreactivity than parasitological and immunological methods [8]. Some mitochondrial genes and schistosome target sequences (e.g., 18S rRNA, SjR2, and 5D) have been used in previous schistosomiasis diagnostic studies [9,10]. A 230-bp DNA fragment of the highly repetitive retrotransposon SjR2 from the *S. japonicum* genome was detected in mouse sera with a specific PCR assay. The DNA fragment was detected 1 week after infection, and was undetectable in week 10 after treatment [6]. A loop-mediated isothermal amplification (LAMP) method and a conventional PCR method were compared by amplifying the target sequence of a recombinant SjR2-pCR 2.1 plasmid template. The LAMP assay was more sensitive than the conventional PCR. The conventional PCR did not detect the DNA fragment in rabbit sera 8–10 weeks after treatment with PZQ, whereas the LAMP assay would take a long duration to achieve this effect [5]. Another LAMP assay also targeting the SjR2 fragment was used to analyze 30 serum samples from *S. japonicum*-infected patients, with a sensitivity of 96.7% [11]. Recombinase polymerase amplification (RPA) combined with a lateral flow dipstick (LFD) assay was developed to detect the *S. haematobium Dra1* gene fragment. This assay is simple, rapid, portable, and sensitive, with a low limit of detection of 100 fg of DNA at 30–45 °C, achieved in 10 min [12]. However, the assay also faces problems such as aerosol pollution during field diagnoses. Therefore, traditional diagnostic methods are still the first choice for field diagnoses at present.

PZQ is the preferred drug for the treatment of schistosomiasis, and the control of the disease relies on mass drug administration (MDA) programs with PZQ. However, there has been no suitable method to evaluate the efficacy of this treatment until now. Furthermore, the prevention and control strategies of schistosomiasis japonica in China have transformed from control to elimination. Therefore, some precise detection methods are gradually required to meet the greater demands in sensitivity and specificity, and evaluation of chemotherapy. A previous qPCR method developed in our laboratory was used to detect schistosomiasis in mice infected with different numbers of cercariae [8]. The detection efficacy and accuracy of the qPCR was then evaluated. The efficacy of PZQ treatment in *S. japonicum*-infected mice was also evaluated with this qPCR method.

## 2. Materials and Methods

### 2.1. Parasites and Animals

*Oncomelania hupensis* snails and BALB/c mice were used to maintain *S. japonicum* throughout its life cycle at the Shanghai Veterinary Research Institute. Cercariae were obtained after schistosome-positive snails were exposed to light to induce shedding and were counted under a microscope. The mice (4–6 weeks old) were then percutaneously infected with different numbers of cercariae for 15 min. Adult schistosomes were collected from the infected mice at 35–42 days post-infection (dpi). Phosphate-buffered saline (PBS) containing 1% sodium citrate was used to perfuse the schistosomes from the hepatic portal veins of the mice, and the schistosomes were collected. Other worms in the mesenteric veins of each mouse were carefully checked, manually removed, and counted.

### 2.2. Sample Collection

Blood samples of mice were collected from the retro-orbital vessels, and they were, respectively, gathered into Eppendorf tubes and EDTA-K_2_ vacuum blood collection tubes. After EDTA-K_2_ vacuum tubes were centrifuged at 1000× *g* for 10 min at 25 °C, the blood samples were separated and the supernatants were collected as plasma samples. After the Eppendorf tubes were centrifuged at 1000× *g* for 10 min at 25 °C, the blood samples were separated and the supernatants were collected as serum samples.

### 2.3. DNA Extraction

DNA was extracted from the mouse plasma samples (0.2 mL/mouse sample) with a Magnetic Serum/Plasma Circulating DNA Maxi Kit (Tiangen Biotech, Beijing, China). The kit uses magnetic beads and a buffer system to separate and purify high-quality free DNA from plasma. The extraction process was performed according to the manufacturer’s protocol. The extracted nucleic acid in the eluent was carefully transferred to a new centrifuge tube and stored at −20 °C.

A TIANamp Genomic DNA Kit (Tiangen Biotech, Beijing, China) was used to extract the schistosome genomic DNA. Adult schistosomes were first cut into small fragments and ground sufficiently on ice. Then, the extraction of schistosome genomic DNA was operated according to the manufacturer’s protocol, and extracted genomic DNA samples were stored at −20 °C.

### 2.4. Preparation of Soluble Egg Antigen (SEA)

The livers were collected from mice infected with *S. japonicum* at 42 dpi and stored at 4 °C for 1 day, homogenized in PBS, and sequentially passed through 60, 80, 120, 160, and 200 mesh screens. The collected solution was centrifuged at 5000× *g* for 10 min and the precipitate was washed three times with PBS. The pellet was resuspended in 100 mL of PBS containing 7.5 U of trypsin and incubated in a constant temperature shaker at 37 °C for 2 h. It was then centrifuged at 5000× *g* for 2 min. The pellet was resuspended in PBS and washed to remove the upper liver paste, as previously described [13]. The purified eggs were checked under a microscope, frozen and thawed three times, and then sonicated with an ultrasonic cell crusher on ice. After centrifugation at 12,000× *g* for 20 min, the supernatant was collected as SEA [13].

### 2.5. The qPCR Assay

The qPCR assay reported in our previous study [8] was performed in a 20 μL reaction mixture containing 2 × ChamQ Universal SYBR^®^ qPCR Master Mix (10 μL; Vazyme, Nanjing, China), forward primer (0.4 μL, 10 µmol/L), reverse primer (0.4 μL, 10 µmol/L), extracted DNA template (4 μL), and double-distilled water (ddH_2_O; 5.2 μL). The no-template control contained ddH_2_O instead of DNA template. The negative control contained DNA template extracted from the sera of uninfected mice. The positive control contained *S. japonicum* genomic DNA as the template in each qPCR assay. The PCR cycling conditions were denaturation at 94 °C for 3 min, followed by 40 cycles of denaturation at 94 °C for 15 s, annealing at 58 °C for 34 s, and extension at 72 °C for 10 s. The results were determined with an amplification curve combined with a melting curve analysis (the characteristic peak).

### 2.6. Detection of Schistosomiasis Japonica in Mice with qPCR

A total of 153 plasma samples were collected from mice artificially infected with 10, 20, 40, ~60, or ~100 cercariae. These mice were used for different experiments in our laboratory, and they were killed between 35 and 42 days after infection. The number of schistosome parasites in each mouse was then counted and recorded, and the plasma samples collected were analyzed with the qPCR assay. A total of 77 mouse plasma samples were collected from 77 mice before they were infected with schistosome.

### 2.7. qPCR Analysis of Infected Mice after PZQ Treatment

Two groups of BALB/c mice were infected with 10 and 40 cercariae, respectively. Then, 23 and 15 BALB/c mice, which were, respectively, infected with 10 and 40 cercariae, were analyzed as positive with qPCR at 35 dpi. And the PCR-positive mice were treated twice with oral PZQ (600 mg/kg bodyweight) at 36 dpi and 42 dpi. The PZQ (Hubei Widely Chemical Technology Co., Ltd., Wuhan, China) was dissolved in 1% carboxymethyl cellulose solution (Sigma-Aldrich, St. Louis, MO, USA) and administrated by gavage. The amount administered was based on the bodyweight of each mouse [14]. Plasma samples from each mouse were collected weekly after treatment with PZQ and analyzed with the qPCR assay. Ten plasma samples from uninfected mice were used as the negative controls. The mice were killed 6 weeks after treatment, and the number of worms in each mouse was counted after perfusion, as described above. The liver of each mouse was excised and the number of eggs per gram (EPG) of liver was calculated as follows.

The liver of each mouse was weighed, recorded, and placed into a 50 mL centrifuge tube. PBS was added to maintain a constant volume of 15 mL. The liver in each 50 mL centrifuge tube was thoroughly ground to a homogeneous solution with a homogenizer. After vortexing, 1 mL of homogenate were removed into a 4 mL Eppendorf tube, and 1 mL of 10% NaOH solution was added. The sample was incubated and the mixture digested at 56 °C for 30 min. An average of three counts per 40 μL of mixture was made, and the number was converted to EPG.

### 2.8. Enzyme-linked Immunosorbent Assay (ELISA) Analysis of Infected Mice after PZQ Treatment

The mouse serum samples collected at the sixth week after treatment with PZQ were analyzed with SEA-ELISA. Ten serum samples from uninfected mice were used as the negative controls. Culture plates (Costar, Washington, DC, USA) were coated with SEA (100 μL, 15 μg/mL), diluted with carbonate bicarbonate buffer (pH 9.6) and incubated overnight at 4 °C. The plates were blocked with 1% gelatin for 1 h at 37 °C. Each serum sample was diluted 1:100 with PBS containing 0.05% Tween 20 (PBST), added to three wells (100 μL/well), and incubated for 1 h at 37 °C. Horseradish–peroxidase-conjugated rabbit anti-mouse immunoglobulin G (IgG) antibody (100 μL, Beyotime, Haimen, China), diluted 1:6000 with PBST, was added to each well and incubated at 37 °C for 45 min. After the wells were washed three times with PBST, 100 μL of 3,3′,5,5′-tetramethylbenzidine dihydrochloride was added to each well, and the reaction was stopped with the addition of 50 μL of 2 M sulfuric acid. The optical density at a wavelength of 450 nm was measured with a microplate reader (BioTek, Winooski, VT, USA). The cutoff value was set to 2.1 times the mean optical density value for the negative control sera [15].

### 2.9. Data Analysis

The 95% confidence intervals (CI) of specificity and sensitivity were calculated using Stata/SE 12.0 (Stata Corp., College Station, TX, USA). Data are expressed as means ± standard deviations (SD).

## 3. Results

### 3.1. Detection of Mouse Schistosomiasis Japonica with the qPCR Assay

In total, 153 plasma samples from mice infected with *S. japonicum* were extracted and analyzed with the qPCR assay. One hundred fifty-two samples tested positive; so, the sensitivity of the qPCR was 99.3% (152/153, 95% CI: 96.41–99.98%). None of the 77 negative plasma samples collected before schistosome infection tested positive; so, the specificity of the qPCR was 100% (77/77, 95% CI: 95.32–100%).

These samples were grouped according to the number of schistosomes present in each mouse, and the positive detection rate was calculated for each group (Table 1). When the number of schistosomes in the mice was less than or equal to 10 (*n* ≤ 10), the positive detection rate was 97.7% (43/44, 95% CI: 87.98–99.94%), and when the worm number was > 10, the positive detection rate was 100% (109/109, 95% CI: 96.67–100%), between 35 and 42 dpi. In the infected mouse that tested negative on the qPCR assay, only two male schistosomes were detected. Thus, the qPCR assay showed high sensitivity and specificity for mice schistosomiasis japonica at 35–42 dpi.

### 3.2. qPCR Detection of Schistosomiasis Japonica in Mice after PZQ Treatment

Twenty-three mice infected with 10 cercariae and 15 mice infected with 40 cercariae were monitored continuously on a weekly basis after PZQ treatment. The results are listed in Table 2 and Table 3. The surveillance results showed that none of the treated mice was identified as *Schistosoma*-positive with the qPCR assay 6 weeks after PZQ treatment (Table 2 and Table 3). It is noteworthy that one mouse (10 cercariae) was identified as *Schistosoma*-negative 1 week after PZQ treatment, and three mice (40 cercariae) were identified as *Schistosoma* negative 3 weeks after PZQ treatment. Even when a sample tested negative at first, it could still test positive on subsequent tests. For example, the first sample of mouse number 1 in Table 2 tested *Schistosoma*-negative 1 week after PZQ treatment, but tested positive 2 and 3 weeks after treatment.

At necropsy, only a few residual male schistosomes were detected in 12 mice. Six of these mice were infected with 10 cercariae, and another six were infected with 40 cercariae. At autopsy, the livers of two mice infected with 10 cercariae showed no granuloma, whereas granuloma was present in the livers of all the other mice.

### 3.3. ELISA Detection of Mice Schistosomiasis Japonica after PZQ Treatment

ELISA detected *Schistosoma* positivity in 65.2% (15/23) of mice infected with 10 cercariae and in 93.3% (14/15) of mice infected with 40 cercariae at the sixth week after PZQ treatment (Table 4 and Table S1). It can be inferred that infection with more cercariae extended the period during which anti-cercarial antibodies were detected after treatment. In one mouse infected with 10 cercariae, the liver showed no granuloma and the mouse tested negative for schistosomiasis 6 weeks after treatment. However, another mouse, in which the liver also showed no granuloma, tested positive for schistosomiasis 6 weeks after PZQ treatment.

## 4. Discussion

In China, extensive comprehensive prevention and control strategies are used to manage schistosomiasis japonica, and the morbidity and transmission of the disease have been maintained at very low levels. MDA programs with PZQ, which is an effective drug for the treatment of schistosomiasis in both humans and domestic animals, is one such effective strategy. Given that several risk factors for disease transmission still exist in areas of low disease prevalence and low-intensity infections, some precise detection methods with high sensitivity and specificity are required to monitor its epidemic status in the future.

A new road map of the World Health Organization (WHO) for controlling or eliminating neglected tropical diseases in 2021–2030 states that accurate, affordable and field-friendly diagnostic and surveillance tools are vital, but these tools are unavailable for the control and elimination of schistosomiasis [16]. Rapid and reliable diagnosis is central to the prevention and control of schistosomiasis [17,18]. Many molecular detection methods have shown both higher sensitivity and specificity and less crossreactivity than traditional methods, such as general PCR, nested PCR, qPCR, digital PCR, and RPA-LFD [8,19]. These laboratory-based molecular diagnostic techniques have been tried to detect schistosome DNA in both the research context and the diagnosis of schistosomiasis, and several methods have potential utility in the detection of schistosomiasis [5,20,21,22].

In this study, a previously published diagnostic qPCR method was used to diagnose schistosomiasis japonica in mice. This method has no crossreactivity with eight other kinds of parasite; meanwhile, it exhibits higher sensitivity and greater specificity than SEA-ELISA in goats [8]. The method also showed high sensitivity (99.3%, 152/153, 95% CI: 96.41–99.98%) and specificity (100%, 77/77, 95% CI: 95.32–100%) in mice. Our results indicate that the diagnostic efficacy of this qPCR is almost identical for both mouse and goat schistosomiasis. In this study, a mouse was infected with 10 cercariae but was identified as *Schistosoma*-negative by qPCR; two male worms were detected at necropsy. Therefore, the schistosomal nucleic acids in the blood might be low when only a small number of schistosomes ultimately survive in the host. Then, a small number of schistosomes or paired worms may be one explanation for the missed diagnosis in this case. Once the worm number in mice exceeded 10, the positive detection rate could be 100% between 35 and 42 dpi.

Although immunological tests have been widely used in many schistosomiasis-endemic areas, current methods cannot be used to evaluate the efficacy of PZQ treatment because IgG antibody levels remain high for a long time, even after the disease is cured. Therefore, given the sensitivity and specificity of molecular diagnostics, their capacity to resolve this problem was evaluated in some previous studies. After PZQ treatment, a PCR assay was used to detect *S. japonicum* DNA in rabbit sera, which became negative at 10 weeks posttreatment [6]. A LAMP assay based on the same target sequence produced a similar result, and the sera became negative at 12 weeks post-treatment [11]. In another similar study, researchers compared the effectiveness of two DNA-based diagnostic techniques, LAMP and PCR, which targeted another sequence, in evaluating a therapy for schistosomiasis. The PCR results were highly consistent with those mentioned in previous studies, but were less sensitive in the detection of schistosomal DNA in drug-treated rabbit sera than the LAMP method [5]. An nPCR method was used to detect a 303-bp sequence of SjCHGCS19 in rabbit sera, which became negative at 17 weeks post-treatment [23].

In the present study, mice infected with 10 or 40 cercariae were all negative for schistosomiasis 6 weeks after PZQ treatment. This was earlier than in previous studies [5,11,23]. This discrepancy may have arisen because the content of the target sequence differed in the blood of the final hosts. After PZQ treatment, no pairs of schistosomes were found in the mice; so, the worms could not lay eggs continuously. The amount of schistosome nucleic acid in the host blood gradually decreased as the worms died, and was not detected in the samples when the target molecule was below the limit of qPCR detection. As shown in Table 2 and Table 3, some mice did not remain continuously negative after they first tested negative. Some mice were first identified as negative in the first 3 weeks after treatment, but became positive again thereafter. This may be because DNA was not continuously released from residual inactive eggs, the residual bodies of dead schistosomes, and/or the tegument shedding of residual schistosomes [5]. Furthermore, although these mice were treated with PZQ, some residual schistosomes could have been found by perfusion (Table 2 and Table 3). This result suggested that after treatment with PZQ, residual inactive eggs and schistosomes could influence the evaluation of the therapeutic efficacy of the drug. In the future, continuous monitoring for a longer period should be undertaken after PZQ treatment.

Although several infected mice were identified with an ELISA as *Schistosoma*-negative 6 weeks after PZQ treatment, most of the other mice were still positive (Table 4). This depended upon the infective dose and the numbers of worms in the mice. If the hosts were infected with more cercariae and more schistosomes survived, the positive rate after PZQ treatment persisted longer. In a previous study, the IgG antibody levels were still high in rabbits 23 weeks after treatment [6]. Therefore, the qPCR assay used here had advantages over immunological tests in evaluating the efficacy of PZQ for schistosomiasis, especially 6 weeks after PZQ treatment. Although the qPCR assay shows higher specificity and sensitivity, a better potential utility for evaluating the treatment efficacy of PZQ and less crossreactivity than traditional methods, this method also presents some shortcomings in point-of-care testing. It will take a long time to extract nucleic acid in plasma, and this process still requires laboratory equipment. In the future, some new molecular diagnostic techniques and DNA extraction methods need to be included to resolve the problem.

## 5. Conclusions

With their greater sensitivity and specificity, molecular techniques have great potential utility in the diagnosis of schistosomiasis japonica and the evaluation of the therapeutic efficacy of its treatment. The techniques also offer insight into the etiological basis of the disease, with the detection of genes of *S. japonicum* in the samples. In theory, proper quarantine procedures can directly determine whether the parasite has been completely expelled from the host after treatment. In the future, it will be imperative to identify more target sequences and combine different molecular diagnostic techniques with suitable target sequences for the accurate diagnosis of schistosomiasis.

## Figures and Tables

**Table 1:** The detection efficacy of qPCR assay for mice schistosomiasis japonica.

Number of Worms (*n*)	Number of Samples	Positive Samples	Positive Rate (95%CI)
*n* ≤ 10	44	43	97.7% (87.98–99.94%)
20 ≥ *n* > 10	32	32	100% (89.11–100%)
40 ≥ *n* > 20	43	43	100% (91.78–100%)
80 ≥ *n* > 40	19	19	100% (82.35–100%)
*n* > 80	15	15	100% (78.20–100%)
Total	153	152	99.3% (96.41–99.98%)

**Table 2:** The detection of each mouse infected with 10 cercariae after PZQ treatment using qPCR assay.

BALB/c Mice	1 w	2 w	3 w	4 w	5 w	6 w	NOW	EPG in Liver
1	−	+	+	−	−	−	0	179 ± 78
2	+	+	+	+	+	−	0	1264 ± 1086
3	+	+	+	−	−	−	0	1772 ± 468
4	+	+	−	−	−	−	0	305 ± 305
5	+	+	+	−	−	−	0	896 ± 784
6	+	+	−	−	−	−	0	1762 ± 549
7	+	+	−	−	−	−	3 ♂	2643 ± 1242
8	+	+	+	−	−	−	0	1766 ± 629
9	+	+	−	−	−	−	0	4349 ± 167
10	−	−	−	−	−	−	0	0
11	−	+	−	−	−	−	2 ♂	0
12	+	−	+	+	−	−	2 ♂	3528 ± 349
13	+	+	−	−	−	−	3 ♂	3746 ± 1782
14	+	−	+	+	+	−	0	465 ± 101
15	+	+	+	−	+	−	0	1355 ± 753
16	+	−	+	−	−	−	0	4111 ± 1135
17	+	+	+	+	−	−	0	3362 ± 776
18	+	+	−	+	+	−	0	1969 ± 731
19	+	−	+	−	−	−	1 ♂	4075 ± 1282
20	+	+	−	+	−	−	3 ♂	3476 ± 1706
21	+	−	+	+	−	−	0	3149 ± 1864
22	+	−	−	−	−	−	0	2848 ± 237
23	+	+	+	−	−	−	0	299 ± 259
Positive rate	87.0%	70.0%	56.5%	30.4%	17.4%	0		

NOW: Number of worms; EPG: Eggs per gram. ‘♂’: male schistosome.

**Table 3:** The detection of each mouse infected with 40 cercariae after PZQ treatment using qPCR assay.

BALB/c Mice	1 w	2 w	3 w	4 w	5 w	6 w	NOW	EPG in Liver
1	+	+	+	−	−	−	0	2993 ± 333
2	+	+	+	−	−	−	3 ♂	3098 ± 406
3	+	+	−	+	+	−	0	3058 ± 172
4	+	+	−	−	−	−	0	1772 ± 145
5	+	+	−	+	−	−	2 ♂	5017 ± 1430
6	+	+	+	−	−	−	2 ♂	7988 ± 2005
7	+	+	+	−	−	−	0	3041 ± 2066
8	+	+	+	−	−	−	1 ♂	2374 ± 936
9	+	+	−	−	−	−	0	2130 ± 1217
10	+	+	+	−	−	−	0	3523 ± 370
11	+	+	−	−	−	−	2 ♂	2773 ± 1001
12	+	+	−	+	+	−	0	1995 ± 794
13	+	+	−	+	−	−	0	2044 ± 1230
14	+	+	−	+	−	−	0	6833 ± 1477
15	+	+	+	−	−	−	1 ♂	4077 ± 1698
Positive rate	100.0%	100.0%	46.7%	33.3%	13.3%	0		

NOW: Number of worms; EPG: Eggs per gram of liver. ‘♂’: male schistosome.

**Table 4:** Comparison of the detection efficacy for mice schistosomiasis japonica at 6 w after PZQ treatment using ELISA and qPCR assay.

	Infected with 10 Cercariae		Infected with 40 Cercariae		No Infection	
	ELISA	qPCR	ELISA	qPCR	ELISA	qPCR
Number of samples	23	23	15	15	10	10
Positive samples	15	0	14	0	0	0
Positive rate	65.20%	0	93.30%	0	0	0
Negative rate	34.80%	100.00%	6.70%	100.00%	\	\

## Data Availability

Not applicable.

## Supplementary Materials

The following supporting information can be downloaded at https://www.mdpi.com/article/10.3390/ani13193068/s1. Table S1: The detection of each mouse infected with 10 or 40 cercariae at the sixth week after PZQ treatment by ELISA.
